# Calcified amorphous tumor of the heart in an adult female: a case report

**DOI:** 10.1186/1752-1947-4-278

**Published:** 2010-08-19

**Authors:** Ruchika Gupta, Milind Hote, Ruma Ray

**Affiliations:** 1 Department of Pathology, All India Institute of Medical Sciences, Ansari Nagar, New Delhi - 110029, India; 2 Department of Cardiothoracic and Vascular Surgery, All India Institute of Medical Sciences, Ansari Nagar, New Delhi - 110029, India

## Abstract

**Introduction:**

Cardiac calcified amorphous tumor is a rare, non-neoplastic intra-cavity cardiac mass composed of calcium deposits in a background of amorphous degenerating fibrinous material. Only a few cases of this rare lesion have been reported in the available literature. Clinico-pathological differentiation of this lesion from calcified atrial myxoma, calcified thrombi or other cardiac neoplasms is extremely difficult; hence pathologic examination is the mainstay of diagnosis. To the best of our knowledge this entity has not been reported in the Indian literature.

**Case presentation:**

A 40-year-old woman of Indian origin presented with progressive dyspnea, fatigue and cough. She was diagnosed as having a calcified right atrial mass. The mass was excised. Histologic examination revealed the mass to be composed of amorphous eosinophilic fibrin with dense calcification. No myxomatous tissue was seen and a final diagnosis of calcified amorphous tumor of the heart was rendered.

**Conclusions:**

Calcified amorphous tumor is a rare cardiac lesion with an excellent outcome following complete surgical removal. Since clinico-radiologic differentiation from other cardiac masses is not possible in most cases, histopathological examination is the only modality for diagnosis. Hence, histopathologists should be aware of this rare entity in the differential diagnoses of cardiac mass.

## Introduction

Cardiac myxomas are the most common primary cardiac tumors, occurring most frequently in left atrium [1]. On the other hand, calcified amorphous tumor (CAT) is a recently described non-neoplastic lesion with a clinical presentation similar to other cardiac masses [2]. Histopathologically, cardiac CAT shows calcified nodules in an amorphous fibrinous background with degeneration and focal chronic inflammation [2]. Excision of the lesion and pathologic examination is mandatory for an accurate diagnosis and differentiation from the more common atrial myxomas. Very few cases of cardiac CAT have been reported in the available English literature [2-7]. To the best of our knowledge, no such case has been reported from the Indian sub-continent.

We describe the case of a right atrial mass, which proved to be a CAT on histopathology. The case is being reported for its rarity and lack of reports in the Indian literature.

## Case presentation

A 40-year-old woman of Indian origin presented with history of gradually worsening breathlessness on exertion, fatigue and non-productive cough for the past six months. There was no significant past or family history. General and systemic examinations were unremarkable.

Routine laboratory investigations were within normal limits. Electrocardiogram and chest radiograph were also unremarkable. Transthoracic echocardiography showed a right atrial mass measuring 3 × 3 × 1.5 cm with focal calcification. A clinical possibility of calcified right atrial myxoma was considered. Our patient underwent cardiac exploration and removal of the mass. Intra-operatively, a calcified mass measuring 3 × 2 × 1.5 cm was noted in the right atrium with multiple sites of attachment to the septum and right atrial wall. The specimen was sent for histopathological examination.

**Figure 1:**
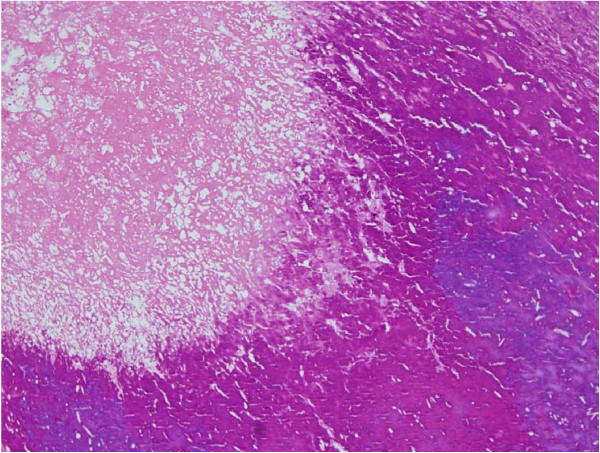
**Photomicrograph showing amorphous eosinophilic fibrinous material along with dense calcification (hematoxylin and eosin (H&E) ×100)**.

**Figure 2:**
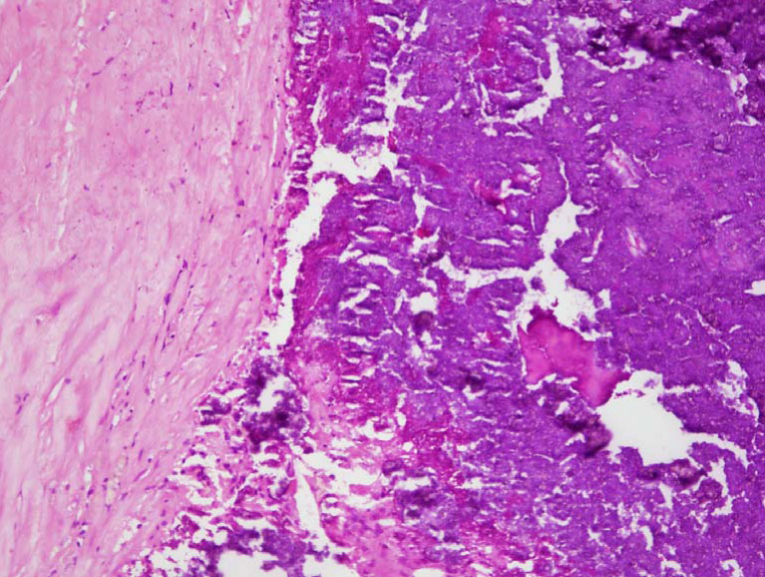
**Photomicrograph demonstrating hyalinized tissue with focal chronic inflammation and a focus of calcification (H&E ×200)**.

We received a single piece of calcified tissue measuring 3 × 2 × 1.5 cm. The lesion was well-circumscribed with focal congestion. The entire tissue was processed for histopathology. Sections showed a lesion composed of a background of eosinophilic amorphous material, possibly degenerated fibrin, with areas of dense calcification and focal chronic inflammation (Figures 1 and 2). Multiple sections were examined and did not reveal any cellular foci of 'myxomatous' tissue. Immunohistochemistry for calretinin was negative. Considering the clinical and histological features, a diagnosis of cardiac calcified amorphous tumor (cardiac CAT) was rendered. Our patient has been doing well during eight months of follow-up.

## Discussion

Primary cardiac tumors are rare and the most common of these are atrial myxomas [1]. However, not all cardiac masses are neoplasms; for instance intra-mural thrombi are great mimics of neoplasms [2,8]. Regardless of the nature of a cardiac mass (neoplastic or not), excision of the lesion is important due to the potential danger of obstruction or embolization and also for accurate diagnosis and therapy. Cardiac CAT is a recently described non-neoplastic intra-cardiac lesion composed of nodules of calcium on a background of amorphous fibrinous material [2]. The rarity of this lesion is borne out by the fact that a 29-year review at the Mayo clinic yielded only 11 such cases [2]. The clinical presentation of cardiac CAT is similar to that of other cardiac masses, i.e. dyspnea, syncope or symptoms related to embolism. Hence, the most frequent clinical impression is a cardiac myxoma, as in our patient. Other clinical differential diagnoses include thrombi, emboli, vegetations and other benign and malignant cardiac tumors [2,9].

Investigations such as echocardiography help in identifying the location, echogenecity and morphology of cardiac masses. In a study of 11 cases, cardiac CATs were described as pedunculated, predominantly left ventricular, diffusely calcified masses on echocardiography [2]. Cardiac myxomas, on the other hand, are mobile left atrial masses (may also involve the right atrium). About 20% of the myxomas may be calcified [10]. Cardiac fibromas may also be calcified; however, they are predominantly left ventricular tumors with an intra-myocardiac location [11]. Other causes of calcification in the heart include chronic renal failure and, rarely, thrombi [2]. In the absence of distinctive clinical and imaging features, a pre-operative differentiation between neoplastic and non-neoplastic lesions remains difficult. Hence, histological diagnosis is the gold standard for a definitive conclusion.

The various histological differential diagnoses for cardiac CAT include myxoma, vegetations, Echinococcus cysts and thrombi. A small fraction of myxomas may calcify and even ossify; hence, adequate sampling is imperative to exclude underlying myxomas [2]. In our case, extensive sampling failed to reveal any myxomatous tissue. Cardiac vegetations are intimately associated with valve leaflets and may rarely calcify. Echinococcosis can be diagnosed by the identification of the cyst wall and presence of scolices [2]. Thrombi may undergo mummification and calcification and mimic cardiac CAT. The absence of predisposing conditions for thrombosis, lack of characteristic laminations of an organizing thrombus and infrequent presence of hemosiderin differentiates CAT from an organizing thrombus [2].

The pathogenesis of cardiac CAT is not certain. However, most authors support the hypothesis that cardiac CAT is an organized and calcified mural thrombus [2-4]. This is supported by the presence of factors predisposing to thrombosis in some patients described in a large series [2]. However, the absence of such predisposing conditions in other patients, such as the present case, suggests that thrombosis may not be the only pathogenetic mechanism for these intriguing cardiac masses.

The majority of the cases reported so far had a benign course after surgical excision of the intra-cardiac mass, although some residual calcium may be seen [2]. One case of a recurrent cardiac CAT in a young patient has also been reported [3]. Another patient, a 60-year-old woman, had a fatal outcome of a cardiac CAT involving right ventricular wall and chordae tendinae of the tricuspid valve [5]. Hence, these patients need to be kept on follow-up after surgical excision with repeat imaging studies in cases with incomplete resection.

## Conclusions

Cardiac CATs are rare intra-cardiac non-neoplastic masses with a presumed thrombotic origin. Since the clinical presentation is similar to other cardiac tumors such as myxoma, surgical excision and histopathologic examination remains the mainstay of an accurate diagnosis.

## Abbreviations

CAT: calcified amorphous tumor

## Consent

Written informed consent was obtained from the patient for publication of this case report and any accompanying images. A copy of the written consent is available for review by the Editor-in-Chief of the journal.

## Competing interests

The authors declare that they have no competing interests.

## Authors' contributions

RG and RR were involved in the signing out of the histopathology report, conducting the literature review and drafting the manuscript. MH was the clinician-in-charge of the daily care of the patient, provided the clinical background, assisted in the drafting and critical review of the manuscript. All the authors have read and approved the final manuscript.
